# Use of and Retention on Video, Telephone, and In-Person Buprenorphine Treatment for Opioid Use Disorder During the COVID-19 Pandemic

**DOI:** 10.1001/jamanetworkopen.2022.36298

**Published:** 2022-10-12

**Authors:** Madeline C. Frost, Lan Zhang, H. Myra Kim, Lewei (Allison) Lin

**Affiliations:** 1 Department of Health Systems and Population Health, University of Washington School of Public Health, Seattle; 2 Health Services Research & Development (HSR&D) Center of Innovation for Veteran-Centered and Value-Driven Care, VA Puget Sound Health Care System, Seattle, Washington; 3 VA Center for Clinical Management Research (CCMR), VA Ann Arbor Healthcare System, Ann Arbor, Michigan; 4 Addiction Center, Department of Psychiatry, University of Michigan, Ann Arbor; 5 Consulting for Statistics, Computing & Analytics Research (CSCAR), University of Michigan, Ann Arbor

## Abstract

**Importance:**

The coronavirus disease 2019 (COVID-19) pandemic prompted policy changes to allow increased telehealth delivery of buprenorphine, a potentially lifesaving medication for opioid use disorder (OUD). It is unclear how characteristics of patients who access different treatment modalities (in-person vs telehealth, video vs telephone) vary, and whether modality is associated with retention—a key indicator of care quality.

**Objectives:**

To compare patient characteristics across receipt of different treatment modalities and to assess whether modality was associated with retention during the year following COVID-19–related policy changes.

**Design, Setting, and Participants:**

This cross-sectional study was conducted in the national Veterans Health Administration. Participants included patients who received buprenorphine for OUD during March 23, 2020, to March 22, 2021. Analyses examining retention were stratified by buprenorphine initiation time (year following COVID-19–related changes; prior to COVID-19–related changes).

**Exposures:**

Patient characteristics; treatment modality (at least 1 video visit, at least 1 telephone visit but no video, only in-person).

**Main Outcomes and Measures:**

Treatment modality; 90-day retention.

**Results:**

Among 17 182 patients, 7094 (41.3%) were aged 30 to 44 years and 6251 (36.4%) were aged 45 to 64 years; 15 835 (92.2%) were male, 14 085 (82.0%) were White, and 16 292 (94.8%) were non-Hispanic; 6547 (38.1%) had at least 1 video visit, 8524 (49.6%) had at least 1 telephone visit but no video visit, and 2111 (12.3%) had only in-person visits. Patients who were younger, male, Black, unknown race, Hispanic, non–service connected, or had specific mental health/substance use comorbidities were less likely to receive any telehealth. Among patients who received telehealth, those who were older, male, Black, non–service connected, or experiencing homelessness and/or housing instability were less likely to have video visits. Retention was significantly higher for patients with telehealth compared with only in-person visits regardless of initiation time (for initiated in year following COVID-19–related changes: adjusted odds ratio [aOR], 1.31; 95% CI, 1.12-1.53; for initiated prior to COVID-19–related changes: aOR, 1.23; 95% CI, 1.08-1.39). Among patients with telehealth, higher retention was observed in those with video visits compared with only telephone for patients who initiated in the year following COVID-19 (aOR, 1.47; 95% CI, 1.26-1.71).

**Conclusions and Relevance:**

In this cross-sectional study, many patients accessed buprenorphine via telephone and some were less likely to have any video visits. These findings suggest that discontinuing or reducing telephone access may disrupt treatment for many patients, particularly groups with access disparities such as Black patients and those experiencing homelessness. Telehealth was associated with increased retention for both new and continuing patients.

## Key Points

**Question:**

Among Veterans Health Administration patients receiving buprenorphine for opioid use disorder in the year following implementation of COVID-19–related telehealth policies, did patient characteristics and retention differ across treatment modalities?

**Findings:**

In this cross-sectional study of 17 182 patients, patients who were younger, male, Black, unknown race, Hispanic, non–service connected, or with certain comorbidities were significantly less likely to receive telehealth; those who were older, male, Black, non–service connected, or experiencing homelessness and/or housing instability were significantly less likely to receive video compared with telephone-only telehealth. Telehealth was positively associated with retention.

**Meaning:**

These findings suggest that discontinuing or reducing telephone-only access may disrupt treatment for groups with access disparities and that telehealth-delivered buprenorphine may support retention.

## Teaser

This cross-sectional study compares Veterans Health Administration patient characteristics across receipt of different treatment modalities for opioid use disorder (OUD) during the year following COVID-19–related policy changes and assesses whether modality was associated with retention.

## Introduction

The COVID-19 pandemic had widespread impacts on health care delivery. Concerns that COVID-19 would disrupt access to potentially lifesaving treatment for people with opioid use disorder (OUD) prompted abrupt federal policy changes in March 2020 to allow increased telehealth delivery of buprenorphine,^1^ a first-line OUD treatment that is known to prevent overdose death and can be prescribed in office-based settings.^2,3^ These changes included eliminating the required initial in-person visit before a patient could be transitioned to telehealth under the public health emergency exception of the Ryan Haight Online Pharmacy Consumer Protection Act.^4^ Additionally, updated guidance from the Drug Enforcement Administration (DEA) and Substance Abuse and Mental Health Services Administration (SAMHSA) allowed—for the first time—buprenorphine visits for new and existing patients to be delivered by telephone (ie, audio-only).^5^ Policy makers and health care leaders are currently debating whether to maintain or roll back policies that have increased flexibility in buprenorphine delivery.^6^ To inform these decisions, it is important to understand the potential relationship of buprenorphine treatment modality (video, telephone, or in-person) with treatment outcomes, as well as the characteristics of patients who are more or less likely to access different modalities.

Prior studies suggest that the rapid expansion of telehealth sustained buprenorphine treatment early in the pandemic.^7,8,9,10^ It is less clear whether patient subgroups who have historically faced disparities in buprenorphine access (eg, Black patients, those experiencing homelessness^11,12,13^) have been more or less likely to access buprenorphine through different modalities (ie, telehealth vs in-person, video vs telephone), and how modalities compare on retention, a critical treatment outcome associated with reductions in overdose.^14,15^ Two studies using national commercial and Medicare claims data found that patients who were older, Black, and had lower income were less likely to initiate buprenorphine for OUD via telehealth compared with in-person; however, these studies did not include other important disparity-related factors such as housing status, did not compare video and telephone modalities, and did not examine treatment retention.^16,17^ Another study conducted in a single OUD treatment program found that older patients had mostly telephone rather than video visits, and that new patients, those with higher education, or those with recent overdose were less likely to have mostly telephone visits.^18^ Other studies conducted in single-clinic settings have compared buprenorphine retention before and after shifting their treatment model from in-person to telehealth; these studies have observed mixed findings and have not compared retention between video and telephone modalities.^19,20,21,22,23^

Studies are needed that compare both key patient characteristics and retention across buprenorphine treatment modalities following COVID-19–related policy changes among a national sample. As the largest OUD treatment provider in the US,^24^ the Veterans Health Administration (VHA) is a particularly important setting in which to examine these questions. VHA has been a leader in expanding telehealth prior to and during the pandemic,^25,26,27^ and rapidly implemented telehealth provision of buprenorphine following COVID-19–related changes in federal policies.^28^ Additionally, the national VHA electronic health record (EHR) includes detailed data allowing for comparison across telephone and video buprenorphine visits. This cross-sectional study aimed to (1) compare patient sociodemographic and clinical characteristics across receipt of different buprenorphine treatment modalities and (2) assess whether treatment modality was associated with retention among VHA patients receiving buprenorphine for OUD in the year following COVID-19–related changes.

## Methods

The VA Ann Arbor Healthcare System institutional review board determined this study to be exempt under Category 4 because it only involves secondary use of data, and a waiver of informed consent was granted because this study is a minimal risk to participants and could not have been practicably conducted otherwise. We followed the Strengthening the Reporting of Observational Studies in Epidemiology (STROBE) reporting guideline for cross-sectional studies.^41^

### Data Source and Study Sample

Data were extracted from the VHA Corporate Data Warehouse, the national repository of VHA electronic health records (EHRs).^29^ The date of COVID-19–related changes affecting buprenorphine delivery was defined as March 23, 2020, the first business day after a national VHA memorandum describing changes in controlled substance prescribing through telehealth was disseminated to clinicians.^30^ The study sample included veterans receiving VHA care aged at least 18 years who filled at least 1 buprenorphine prescription in the year following these changes (March 23, 2020, to March 22, 2021), with an OUD diagnosis documented in the 365 days prior to or including the date of their first buprenorphine fill during this year. OUD was defined using *International Statistical Classification of Diseases, Tenth Revision, Clinical Modification (ICD-10-CM)* codes (eTable 1 in the Supplement).

We expected that the association between treatment modality and retention may differ based on the timing of patients’ buprenorphine initiation relative to COVID-19–related changes, as the potential association of modality with retention may be influenced by how long a patient had been receiving buprenorphine previously. Therefore, when examining the association between modality and retention, we stratified the study sample into 2 mutually exclusive subsamples: (1) initiated a new buprenorphine treatment episode during the year following COVID-19–related changes (no fills December 23, 2019, to March 22, 2020, ie, the 90 days prior to COVID-19–related changes) and (2) initiated prior to COVID-19–related changes (at least 1 fill between December 23, 2019, and March 22, 2020).

### Measures

Buprenorphine treatment modality during the year following COVID-19–related changes was a mutually exclusive categorical variable defined as video (≥1 video visit), telephone (≥1 telephone visit, but no video visits), or in-person (only in-person visits). Modality was defined using VHA visit codes, *Current Procedural Terminology (CPT) *codes, and *ICD-10-CM* codes corresponding to video, telephone, or in-person modalities associated with a clinician who wrote the patient a buprenorphine prescription.^28,31^ Variable definitions involving *ICD-10-CM*,* CPT*, and/or visit codes are described in eTable 1 in the Supplement.

Patient sociodemographic and clinical characteristics were captured based on EHR documentation within the year prior to the patient’s first buprenorphine fill during the year following COVID-19–related changes (including the day of the fill). These included age group, sex, race, ethnicity, VHA eligibility status, rurality of patient residence based on Rural-Urban Commuting Area codes,^32^ homelessness and/or housing instability based on *ICD-10-CM* and/or visit codes,^33^ substance use disorders and mental health diagnoses including alcohol use disorder, cannabis use disorder, stimulant use disorder (cocaine or amphetamine), other drug use disorder (sedative, hallucinogen, inhalant, and/or other psychoactive substance), depressive disorder, posttraumatic stress disorder (PTSD), other anxiety disorder, serious mental illness (bipolar, schizophrenia and/or psychosis), and number of Elixhauser comorbid conditions.^34^ VHA eligibility status is a measure of eligibility for VHA services and an indirect measure of socioeconomic status, both of which may impact OUD treatment modality. Service connection reflects eligibility for lower cost or cost-free health care due to disability connected to military service (and may reflect reduced earning capacity related to disability), and being non-service connected reflects eligibility for VHA services for other reasons such as low financial resources.^35,36^

Buprenorphine treatment retention was a binary variable defined as having at least 90 days of buprenorphine coverage with no more than a 30-day gap in coverage from the patient’s first buprenorphine fill during the year following COVID-19–related changes. This definition has been used in prior studies examining buprenorphine retention in the VHA,^37^ and allows for standardized comparison of retention regardless of whether a patient initiated buprenorphine during or prior to the study period.

### Statistical Analysis

There were 2 analytic goals: first, to compare patient sociodemographic and clinical characteristics across buprenorphine treatment modalities, and second, to examine the association between treatment modality and retention. We first compared patients who received any telehealth (video or telephone) visits with those who received only in-person visits. In the subset of patients who received any telehealth, we further compared patients who received any video visits with those who received only telephone visits. The latter comparison was of particular interest because the question of whether to maintain telephone-only access to buprenorphine is currently being debated.^6^

We compared patient characteristics across modality using χ^2^ tests. We then fit 2 generalized linear models with generalized estimating equation (GEE) and a logit link function to model (1) any telehealth vs only in-person visits as the outcome among all patients and (2) any video vs only telephone visits as the outcome among those who received any telehealth. Models were clustered by facility to account for correlation of data within the same facility,^38,39^ and included all patient characteristics described previously as independent variables to examine the independent association of each with modality.

When examining retention across buprenorphine treatment modalities, we stratified analyses by the 2 subsamples described previously (initiated during the year following COVID-19–related changes, initiated prior to changes). Prevalence of 90-day retention was descriptively compared across modality using χ^2^ tests. We then fit 2 generalized linear models with GEE and a logit link function, both modeling 90-day retention as the outcome. One model was fit among all patients and compared those who received any telehealth with those who received only in-person visits; the second model was fit among patients who received any telehealth and compared those who received any video visits with those who received only telephone visits. The models were clustered by VHA facility to account for correlation of data within the same facility^38,39^ and were adjusted for all patient sociodemographic and clinical characteristics previously described which we considered potential confounders of the association between modality and retention.

Analyses were conducted using SAS Enterprise Guide Software version 7.1 (SAS Institute).^40^ *P* values less than .05 from 2-sided significance tests were considered statistically significant.

## Results

Among 17 182 patients, 7094 (41.3%) were aged 30 to 44 years and 6251 (36.4%) were aged 45 to 64 years; 15 835 (92.2%) were male, 14 085 (82.0%) were White, and 16 292 (94.8%) were non-Hispanic. There were 6547 patients (38.1%) who had at least 1 video visit, 8524 (49.6%) had at least 1 telephone visit but no video visit, and 2111 (12.3%) had only in-person visits.

### Comparison of Patient Characteristics Across Treatment Modalities

Descriptive comparisons of patient characteristics are presented in Table 1. Among all patients, those with any telehealth visits were more frequently older (eg, among those with any telehealth visits: 3115 [20.7%] were aged at least 65 years; and among those with only in-person visits: 352 [16.7%] were aged at least 65 years), female (1209 patients [8.0%] among those with any telehealth visits vs 138 patients [6.5%] among those with only in-person visits) and White (12 408 patients [82.3%] vs 1677 patients [79.4%]). Compared with patients who received any telehealth, those with only in-person visits more frequently had homelessness and/or housing instability (770 patients [36.5%] among those with only in-person visits vs 3507 patients [23.3%] among those with any telehealth visits) and substance use and mental health disorders (eg, alcohol use disorder: 957 patients [45.3%] vs 5173 patients [34.3%]). Among patients with any telehealth visits, those with any video visits were more frequently younger (eg, among those with any video visits: 3014 [46.0%] were aged 30 to 44 years; and among those with only telephone visits: 3143 [36.9%] were aged 30 to 44 years), female (608 patients [9.3%] among those with any video visits vs 601 patients [7.1%] among those with only telephone visits), White (5511 patients [84.2%] vs 6897 patients [80.9%]), service connected 50% to 100% (3671 patients [56.1%] vs 4134 patients [48.5%]), and diagnosed with PTSD (3245 patients [49.6%] vs 3854 patients 45.2%), while those with only telephone visits more frequently had homelessness and/or housing instability (2080 patients [24.4%] among those with only telephone visits vs 1427 patients [21.8%] among those with any video visits) and a larger number of Elixhauser comorbid conditions (eg, among those with only telephone visits: 4491 [52.7%] had at least 3 comorbidities; and among those with any video visits: 3138 [47.9%] had at least 3 comorbidities).

**Table 1.** Descriptive Comparison of Patient Characteristics Across Treatment Modalities Among VHA Patients Who Received Buprenorphine for OUD in the Year Following COVID-19–Related Changes

Characteristic	All patients (n = 17 182)			Patients with any telehealth visits (n = 15 071)		
	No. (%)		*P* value^a^	No. (%)		*P* value^a^
	≥1 Video or telephone visit	Only in-person visits		≥1 Video visit	≥1 Telephone visit; no video	
Age group						
18-29	299 (2.0)	71 (3.4)	<.001	147 (2.2)	152 (1.8)	<.001
30-44	6157 (40.9)	937 (44.4)		3014 (46.0)	3143 (36.9)	
45-64	5500 (36.5)	751 (35.6)		2313 (35.3)	3187 (37.4)	
≥65	3115 (20.7)	352 (16.7)		1073 (16.4)	2042 (24.0)	
Sex						
Female	1209 (8.0)	138 (6.5)	.02	608 (9.3)	601 (7.1)	<.001
Male	13 862 (92.0)	1973 (93.5)		5939 (90.7)	7923 (92.9)	
Race						
American Indian/Alaska Native	130 (0.9)	15 (0.7)	.004	61 (0.9)	69 (0.8)	<.001
Asian/Pacific Islander	139 (0.9)	21 (1.0)		71 (1.1)	68 (0.8)	
Black	1646 (10.9)	290 (13.7)		575 (8.8)	1071 (12.6)	
White	12 408 (82.3)	1677 (79.4)		5511 (84.2)	6897 (80.9)	
Unknown	748 (5.0)	108 (5.1)		329 (5.0)	419 (4.9)	
Ethnicity						
Hispanic	766 (5.1)	124 (5.9)	.12	351 (5.4)	415 (4.9)	.17
Non-Hispanic	14 305 (94.9)	1987 (94.1)		6196 (94.6)	8109 (95.1)	
VA eligibility status						
Non-service connected	4631 (30.7)	661 (31.3)	.86	1774 (27.1)	2857 (33.5)	<.001
Service connection <50%	2635 (17.5)	364 (17.2)		1102 (16.8)	1533 (18.0)	
Service connection 50%-100%	7805 (51.8)	1086 (51.4)		3671 (56.1)	4134 (48.5)	
Rurality						
Urban	12 888 (85.5)	1826 (86.5)	.17	5609 (85.7)	7279 (85.4)	.77
Large rural	1096 (7.3)	147 (7.0)		477 (7.3)	619 (7.3)	
Small/isolated rural	801 (5.3)	91 (4.3)		334 (5.1)	467 (5.5)	
Unknown	286 (1.9)	47 (2.2)		127 (1.9)	159 (1.9)	
Homelessness/housing instability	3507 (23.3)	770 (36.5)	<.001	1427 (21.8)	2080 (24.4)	<.001
Alcohol use disorder	5173 (34.3)	957 (45.3)	<.001	2226 (34.0)	2947 (34.6)	.46
Cannabis use disorder	1865 (12.4)	424 (20.1)	<.001	793 (12.1)	1072 (12.6)	.39
Stimulant use disorder	4301 (28.5)	951 (45.0)	<.001	1852 (28.3)	2449 (28.7)	.55
Other drug use disorder^b^	3302 (21.9)	799 (37.8)	<.001	1452 (22.2)	1850 (21.7)	.49
Depressive disorder	7850 (52.1)	1228 (58.2)	<.001	3433 (52.4)	4417 (51.8)	.45
Posttraumatic stress disorder	7099 (47.1)	1102 (52.2)	<.001	3245 (49.6)	3854 (45.2)	<.001
Anxiety disorder	5940 (39.4)	949 (45.0)	<.001	2619 (40.0)	3321 (39.0)	.19
Serious mental illness^c^	2391 (15.9)	472 (22.4)	<.001	1090 (16.6)	1301 (15.3)	.02
No. of Elixhauser comorbidities (excluding OUD)						
0	1726 (11.5)	159 (7.5)	.15	782 (11.9)	944 (11.1)	<.001
1	2788 (18.5)	303 (14.4)		1285 (19.6)	1503 (17.6)	
2	2928 (19.4)	340 (16.1)		1342 (20.5)	1586 (18.6)	
≥3	7629 (50.6)	1309 (62.0)		3138 (47.9)	4491 (52.7)	

^a^ *P* value from χ^2^ test.

^b^ Includes sedative, hallucinogen, inhalant, and/or other psychoactive substance.

^c^ Includes bipolar disorder, psychosis, and/or schizophrenia.

Results from the 2 adjusted GEE models reporting associations between patient characteristics and buprenorphine treatment modalities are presented in Table 2. Among all patients, those who were older (aged at least 65 years vs 18 to 29 years: adjusted odds ratio [aOR], 1.94; 95% CI, 1.40-2.68) or service connected 50% to 100% (vs non-service connected: aOR, 1.24; 95% CI, 1.09-1.41) were more likely to have any telehealth visits compared with only in-person visits. Those who were male (aOR, 0.74; 95% CI, 0.61-0.91), Black (vs White: aOR, 0.71; 95% CI, 0.61-0.84) or unknown race (vs White; aOR, 0.77; 95% CI, 0.62-0.97), Hispanic ethnicity (aOR, 0.75; 95% CI, 0.59-0.94), or had alcohol use disorder (aOR, 0.84; 95% CI, 0.74-0.95), stimulant use disorder (aOR, 0.64; 95% CI, 0.57-0.72), other drug use disorder (aOR, 0.68; 95% CI, 0.60-0.77), or serious mental illness (aOR, 0.81; 95% CI, 0.71-0.92) were less likely to have any telehealth visits. In the subset of patients who had any telehealth visits, those who were service connected less than 50% (vs non–service connected: aOR, 1.13; 95% CI, 1.02-1.26) or 50% to 100% (vs non–service connected: aOR, 1.28; 95% CI, 1.16-1.40) were more likely to have any video visits compared with only telephone visits. Those who were older (eg, aged at least 65 years vs 18 to 29 years: aOR, 0.53; 95% CI, 0.41-0.70), male (aOR, 0.81; 95% CI, 0.71-0.92), Black (vs White: aOR, 0.73; 95% CI, 0.64-0.83), or had homelessness and/or housing instability (aOR, 0.81; 95% CI, 0.73-0.89) were less likely to have any video visits.

**Table 2.** Associations Between Patient Characteristics and Treatment Modality Among VHA Patients Who Received Buprenorphine for OUD in the Year Following COVID-19–Related Changes

Characteristic	aOR^a^ (95% CI) for any telehealth vs in-person only among all patients (n = 17 182)	aOR^a^ (95% CI) for any video vs telephone only among patients with any telehealth visits (n = 15 071)
Age group, y		
18-29	1 [Reference]	1 [Reference]
30-44	1.45 (1.08-1.95)	1.01 (0.78-1.30)
45-64	1.77 (1.31-2.39)	0.78 (0.60-1.01)
≥65	1.94 (1.40-2.68)	0.53 (0.41-0.70)
Male sex	0.74 (0.61-0.91)	0.81 (0.71-0.92)
Race		
White	1 [Reference]	1 [Reference]
American Indian/Alaska Native	1.07 (0.59-1.94)	0.98 (0.67-1.44)
Asian/Pacific Islander	0.76 (0.46-1.26)	1.07 (0.74-1.54)
Black	0.71 (0.61-0.84)	0.73 (0.64-0.83)
Unknown	0.77 (0.62-0.97)	0.95 (0.80-1.12)
Hispanic ethnicity	0.75 (0.59-0.94)	0.90 (0.75-1.07)
VA eligibility status		
Non-service connected	1 [Reference]	1 [Reference]
Service connection		
<50%	1.11 (0.96-1.30)	1.13 (1.02-1.26)
50%-100%	1.24 (1.09-1.41)	1.28 (1.16-1.40)
Rurality		
Urban	1 [Reference]	1 [Reference]
Large rural	1.13 (0.91-1.38)	1.02 (0.88-1.18)
Small/isolated rural	1.25 (0.97-1.62)	0.97 (0.82-1.15)
Unknown	0.88 (0.62-1.25)	0.91 (0.69-1.21)
Homelessness/housing instability	1.08 (0.95-1.23)	0.81 (0.73-0.89)
Alcohol use disorder	0.84 (0.74-0.95)	0.98 (0.90-1.08)
Cannabis use disorder	0.88 (0.77-1.02)	0.97 (0.86-1.08)
Stimulant use disorder	0.64 (0.57-0.72)	0.97 (0.89-1.07)
Other drug use disorder	0.68 (0.60-0.77)	1.02 (0.93-1.13)
Depressive disorder	0.92 (0.82-1.04)	1.04 (0.96-1.13)
Posttraumatic stress disorder	0.93 (0.82-1.04)	0.97 (0.89-1.05)
Anxiety disorder	1.04 (0.94-1.17)	1.00 (0.92-1.08)
Serious mental illness	0.81 (0.71-0.92)	1.05 (0.95-1.16)
No. of Elixhauser comorbidities (excluding OUD)		
0	1 [Reference]	1 [Reference]
1	0.98 (0.79-1.22)	1.10 (0.95-1.26)
2	1.07 (0.85-1.34)	1.10 (0.95-1.27)
≥3	0.95 (0.75-1.20)	1.08 (0.93-1.25)

Abbreviations: aOR, adjusted odds ratio; OUD, opioid use disorder; VHA, Veterans Health Administration.

^a^ Adjusted for all other covariates in the model.

### Association Between Treatment Modality and Retention

Among 4338 patients who initiated a new buprenorphine treatment episode during the year following COVID-19–related changes, 1318 (30.4%) were retained at least 90 days from initiation. Among 12 844 patients who initiated buprenorphine prior to COVID-19–related changes, 6482 (50.5%) were retained at least 90 days from their first buprenorphine fill during the year following COVID-19–related changes.

Across both subsamples, retention was significantly higher among those who had any telehealth visits compared with those who had only in-person visits (for initiated in year following COVID-19–related changes: aOR, 1.31; 95% CI, 1.12-1.53; for initiated prior to COVID-19–related changes: aOR, 1.23; 95% CI, 1.08-1.39) (Table 3, Figure; eTable 2 in the Supplement). In the subsample of patients who initiated buprenorphine during the year following COVID-19–related changes, retention was significantly higher in those who had any video visits compared with only telephone visits among those who received any telehealth (aOR, 1.47; 95% CI, 1.26-1.71). Retention did not significantly differ between those who received any video compared with only telephone visits among patients who initiated buprenorphine prior to COVID-19–related changes.

**Table 3.** Descriptive Comparison of Retention Across Treatment Modalities Among VHA Patients Who Received Buprenorphine for OUD in the Year Following COVID-19–Related Changes

	All patients (n = 17 182)			Patients with any telehealth visits (n = 15 071)		
	≥1 Video or telephone visit, No. (%)	Only in-person visits, No. (%)	*P* value^a^	≥1 Video visit, No. (%)	≥1 Telephone visit; no video, No. (%)	*P* value^a^
**Initiated in year following COVID-19 (n = 4338 among all patients)**						
Retained ≥90 d	1024 (32.2)	294 (25.4)	<.001	565 (37.2)	459 (27.7)	<.001
Not retained ≥90 d	2156 (67.8)	864 (74.6)		955 (62.8)	1201 (72.3)	
**Initiated prior to COVID-19 (n = 12 844 among all patients)**						
Retained ≥90 d	6072 (51.1)	410 (43.0)	<.001	2610 (51.9)	3462 (50.4)	.09
Not retained ≥90 d	5819 (48.9)	543 (57.0)		2417 (48.1)	3402 (49.6)	

^a^ *P* value from χ^2^ test.

**Figure.**
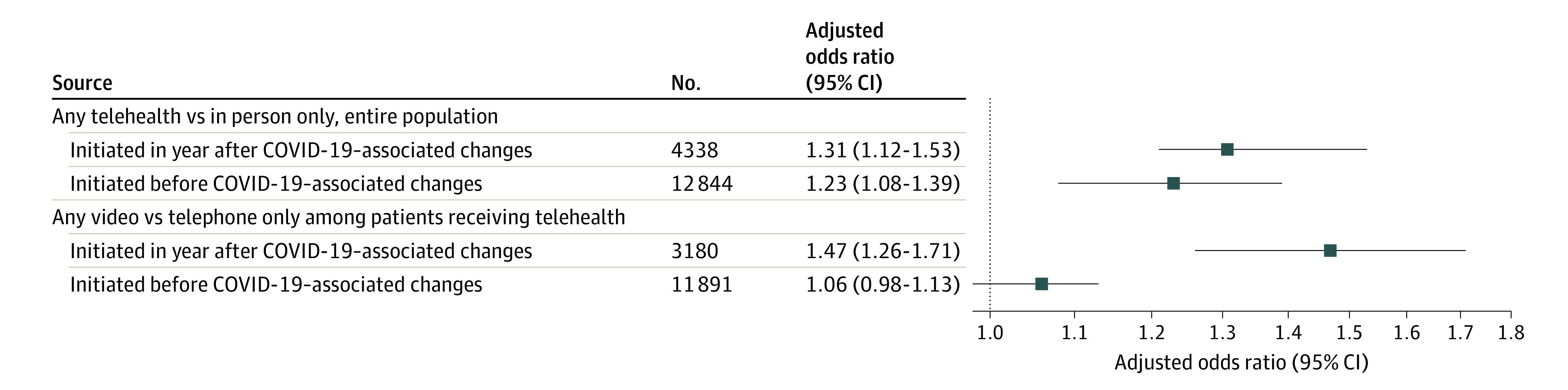
Association Between Modality and 90-Day Retention Among VHA Patients Who Received Buprenorphine Following COVID-19–Related Changes

## Discussion

This cross-sectional study of VHA patients who received buprenorphine for OUD during the year following COVID-19–related policy changes found that some groups of patients were less likely to receive any telehealth (including those who were younger, male, Black, unknown race, Hispanic, non–service connected, or had alcohol use disorder, stimulant use disorder, other drug use disorder, or serious mental illness), and among those who received telehealth some groups were less likely to have any video visits (including those who were older, male, Black, non–service connected, or experiencing homelessness and/or housing instability). Overall, receiving telehealth-delivered buprenorphine was associated with 90-day retention.

Most patients receiving buprenorphine for OUD following COVID-19–related policy changes either initiated or continued this treatment via telehealth, therefore revoking policies that have increased buprenorphine telehealth access may disrupt care for many patients. However, as some groups were less likely to receive telehealth, additional efforts are needed to improve equitable buprenorphine access in addition to maintaining telehealth policies. Moreover, some groups with disparities in buprenorphine access documented prior to COVID-19 (eg, Black patients, those experiencing homelessness^11,12,13^) were more likely to access buprenorphine telehealth through telephone visits only. Racism and socioeconomic inequality have profoundly impacted OUD treatment and resulted in unequal buprenorphine access.^42,43^ Maintaining telephone-only access may be one means of promoting equity in buprenorphine receipt, although broader efforts to address disparities are needed.^44,45^ At the same time, these findings suggest that receipt of video telehealth may support retention among new patients. Rather than revoking telephone-only access—which would likely disrupt treatment for many patients and particularly groups with existing disparities—efforts to increase video access may be needed. The VHA has invested in supporting video telehealth access, including providing video-enabled internet-connected devices to patients and connecting patients to discounted internet services and community telehealth sites.^46^ Additional efforts beyond the health care system level are likely needed, including policies that address disparities in broadband access.^47^

Receiving telehealth was positively associated with retention—regardless of when patients initiated buprenorphine relative to COVID-19–related changes—therefore, maintaining telehealth may contribute to improved retention for both new and continuing patients. Telehealth may address known barriers to continuing treatment, such as distance to care.^48^ Patients who initiated after COVID-19–related changes had a lower proportion of 90-day retention compared with those who initiated prior to COVID-19–related changes, which is not surprising because prior duration on buprenorphine is likely associated with future retention. Overall rates of retention during the year following COVID-19–related changes were low, and it is possible that broader social and economic instability related to the pandemic negatively impacted retention even if telehealth supported retention for patients who received it. Additional efforts to help patients remain in treatment beyond expanded telehealth are needed.

There have been no published randomized clinical trials examining effectiveness of telehealth for OUD and this is one of the first studies to compare both key patient characteristics and retention across buprenorphine treatment modalities (including comparison of video vs telephone modalities) in a national sample in the context of COVID-19–related policy changes. However, more research is needed to inform policies and interventions to improve OUD care. Future research should examine whether the association of modality with retention differs across patient characteristics to consider whether different groups of patients tend to benefit more from telehealth-delivered buprenorphine. Studies should also examine the association of modality with other elements of treatment quality, including adverse outcomes such as overdose events. The practices of individual clinicians will impact whether patients are offered buprenorphine telehealth (regardless of policies allowing these modalities), and several recent studies examining clinicians’ experiences have found variability in their comfort and interest in continuing to offer telehealth.^49,50,51,52,53^ Additional studies with clinicians providing buprenorphine beyond the COVID-19 context are needed to identify and address barriers to continuing telehealth. Research should also continue to examine patient experiences and preferences related to buprenorphine treatment modality.^54,55,56^

### Limitations

This cross-sectional study had some limitations. It was an exploratory study aiming to describe patient characteristics across telehealth and in-person treatment modalities and to examine associations between treatment modality and retention. These associations have important implications for forthcoming policy decisions; however, results from this study cannot be interpreted as causal. A randomized trial is needed to determine whether telehealth directly improves retention and other treatment outcomes. Modality type may have been misclassified for some visits if there was variation in how visits were coded across clinicians, and these data do not capture buprenorphine that may have been received outside of the VHA. Results from stratified analyses may have been sensitive to how initiation timing categories were defined, and we were unable to examine further heterogeneity of associations based on varying total length in buprenorphine treatment prior to COVID-19–related changes. Additionally, although we accounted for facility-level correlation of data, this study does not examine the potential role of clinician- and clinic-level variation in receipt of different treatment modalities, which should be examined in future research. Furthermore, this study was unable to assess potential mechanisms underlying differences in treatment modality across patient groups, which could involve multiple factors such as clinician decision-making, patients’ preferences, and/or contextual factors. More research is needed assessing the perspectives of patients and clinicians to understand these mechanisms. Finally, while it is essential to understand telehealth-delivered buprenorphine care in the VHA given the large number of patients with OUD served by this system, these findings may have limited generalizability in other health care settings.

## Conclusions

This study found that among VHA patients who received buprenorphine for OUD during the year following COVID-19–related policy changes, most had telehealth visits, and most who received telehealth had telephone but no video visits. Patients who were younger, male, Black, unknown race, Hispanic, non–service connected, or had specific mental health/substance use comorbidities were less likely to receive any telehealth. Among patients who received any telehealth, those who were older, male, Black, non–service connected, or had homelessness and/or housing instability were less likely to have video visits. Telehealth receipt was associated with 90-day retention regardless of when patients had initiated buprenorphine relative to the implementation of COVID-19–related policies. Among those who had initiated during the year following COVID-19–related changes and received telehealth, having any video visits was associated with 90-day retention. Policy makers and clinical leaders should carefully consider the potential impacts of forthcoming decisions related to buprenorphine telehealth policies. Discontinuation or reduction of telehealth availability may disrupt treatment for many patients, and discontinuation or reduction of telephone-only access may have an outsized effect on groups who have historically faced disparities in buprenorphine access. Maintaining video and telephone telehealth modalities and improving access to video telehealth may contribute to improved retention.

## Notes

Supplement. **eTable 1.** Definitions of Measures Using Diagnostic Codes and/or Clinic Visit Codes **eTable 2.** Association Between Treatment Modality and 90-Day Retention Among VHA Patients Who Received Buprenorphine for OUD in the Year Following COVID-19-Related Changes
