# Supplementary material for: Use of and Retention on Video, Telephone, and In-Person Buprenorphine Treatment for Opioid Use Disorder During the COVID-19 Pandemic
Source: JAMA Netw Open. 2022 Oct 12;5(10):e2236298. doi: 10.1001/jamanetworkopen.2022.36298 (PMC9557869; doi:10.1001/jamanetworkopen.2022.36298)
Supplement: Supplement. — eTable 1. Definitions of Measures Using Diagnostic Codes and/or Clinic Visit Codes eTable 2. Association Between Treatment Modality and 90-Day Retention Among VHA Patients Who Received Buprenorphine for OUD in the Year Following COVID-19-Related Changes [file jamanetwopen-e2236298-s001.pdf]

Supplemental Online Content

Frost MC, Zhang L, Kim HM, Lin LA. Use of and retention on video, telephone, and in-person buprenorphine treatment for opioid use disorder during the COVID-19 pandemic. *JAMA Netw Open.* 2022;5(10):e2236298. doi:10.1001/jamanetworkopen.2022.36298

eTable 1. Definitions of Measures Using Diagnostic Codes and/or Clinic Visit Codes

eTable 2. Association Between Treatment Modality and 90-Day Retention Among VHA Patients Who Received Buprenorphine for OUD in the Year Following COVID-19-Related Changes

This supplemental material has been provided by the authors to give readers additional information about their work.

© 2022 Frost MC et al. *JAMA Network Open.*

eTable 1. Definitions of measures using diagnostic codes and/or clinic visit codes

Measure	Definition
Opioid use disorder	ICD-10-CM codes:<br>F11*
Video telehealth modality	Clinic visit codes (secondary):<br>690, 692, 693, 699, 644, 645, 136, 137, 444, 445, 446, 447, 440, 708, 723, 724, 490, 491, 179, 679, 648<br>Or with CPT modifier = GT/95
Telephone telehealth modality	CPT codes:<br>99441, 99442, 99443<br>or<br>Clinic visits codes (primary):<br>545, 527, 338 (or other codes with "phone' in the name which including: 103, 147, 148, 169, 178, 181, 182, 199, 216, 221, 229, 324, 325, 326, 424, 425, 428, 441, 451, 452, 453, 454, 462, 463, 476, 526, 527, 528, 530, 536, 537, 542, 543, 544, 545, 546, 579, 584, 597, 609, 611, 686, 729, 801, 802, 803)
Homelessness and/or housing instability	ICD-10-CM codes:<br>Z59.0, Z59.1, Z59.3, Z59.8, Z59.9<br>Clinic visit codes:<br>504, 507, 508, 511, 522, 528, 529, 530, 555, 556, 590, 76, 453, 455, 458, 460, 501
Alcohol use disorder	ICD-10-CM codes:<br>F10*
Stimulant use disorder	ICD-10-CM codes:<br>F14*, F15*
Cannabis use disorder	ICD-10-CM codes:<br>F12*
Other drug use disorder (sedative, hallucinogen, inhalant and/or other psychoactive substance)	ICD-10-CM codes:<br>F13*, F16*, F18*, F19*
Depressive disorder	ICD-10-CM codes:<br>F32*, F33*
Post-traumatic stress disorder	ICD-10-CM codes:<br>F43.1*
Other anxiety disorder	ICD-10-CM codes:<br>F06.4, F40*, F41*, F42*, F43*, F45.2*, R45.7
Serious mental illness (bipolar disorder, psychosis and/or schizophrenia)	ICD-10-CM codes:<br>F20*, F25*, F22, F23, F24, F28, F29, F53.1, F06.0, F06.2, F06.3*, F30*, F31*

ICD-10-CM = International Classification of Diseases, 10th Revision, Clinical Modification; CPT = Current Procedural Terminology

* Indicates inclusion of all sub-codes under parent code

© 2022 Frost MC et al. *JAMA Network Open.*

eTable 2. Association between treatment modality and 90-day retention among VHA patients who received buprenorphine for OUD in the year following COVID-19-related changes

	All patients (N=17,182)		Patients with any telehealth visits (N=15,071)	
	aOR[a] for retained ≥90 days (predictor: any telehealth vs. in-person only)	95% CI	aOR[a] for retained ≥90 days (predictor: any video vs. telephone only)	95% CI
Initiated in year following COVID (N=4,338 among all patients)	1.31	(1.12, 1.53)	1.47	(1.26, 1.71)
Initiated prior to COVID (N=12,844 among all patients)	1.23	(1.08, 1.39)	1.06	(0.98, 1.13)

aOR = adjusted odds ratio; CI = confidence interval; OUD = opioid use disorder; VHA = Veterans Health Administration

[a]Adjusted for age, sex, race, ethnicity, VHA eligibility status, rurality of patient residence, homelessness and/or housing instability, alcohol use disorder, cannabis use disorder, stimulant use disorder, other drug use disorder (sedative, hallucinogen, inhalant and/or other psychoactive substance), depressive disorder, post-traumatic stress disorder, other anxiety disorder, serious mental illness (bipolar disorder, psychosis and/or schizophrenia), and number of Elixhauser comorbidities (excluding OUD).

© 2022 Frost MC et al. *JAMA Network Open.*
